# A Chromosome-Scale Assembly of the Wheat Leaf Rust Pathogen *Puccinia triticina* Provides Insights Into Structural Variations and Genetic Relationships With Haplotype Resolution

**DOI:** 10.3389/fmicb.2021.704253

**Published:** 2021-07-29

**Authors:** Jing Qin Wu, Long Song, Yi Ding, Chongmei Dong, Mafruha Hasan, Robert F. Park

**Affiliations:** Plant Breeding Institute, School of Life and Environmental Sciences, Faculty of Science, The University of Sydney, Sydney, NSW, Australia

**Keywords:** wheat leaf rust, chromosome-scale assembly, mating type genes, avirulence gene, phylogeny

## Abstract

Despite the global economic importance of the wheat leaf rust pathogen *Puccinia triticina* (*Pt*), genomic resources for *Pt* are limited and chromosome-level assemblies of *Pt* are lacking. Here, we present a complete haplotype-resolved genome assembly at a chromosome-scale for *Pt* using the Australian pathotype 64-(6),(7),(10),11 (Pt64; North American race LBBQB) built upon the newly developed technologies of PacBio and Hi-C sequencing. PacBio reads with ∼200-fold coverage (29.8 Gb data) were assembled by Falcon and Falcon-unzip and subsequently scaffolded with Hi-C data using Falcon-phase and Proximo. This approach allowed us to construct 18 chromosome pseudomolecules ranging from 3.5 to 12.3 Mb in size for each haplotype of the dikaryotic genome of Pt64. Each haplotype had a total length of ∼147 Mb, scaffold *N*_50_ of ∼9.4 Mb, and was ∼93% complete for BUSCOs. Each haplotype had ∼29,800 predicted genes, of which ∼2,000 were predicted as secreted proteins (SPs). The investigation of structural variants (SVs) between haplotypes A and B revealed that 10% of the total genome was spanned by SVs, highlighting variations previously undetected by short-read based assemblies. For the first time, the mating type (MAT) genes on each haplotype of Pt64 were identified, which showed that MAT loci *a* and *b* are located on two chromosomes (chromosomes 7 and 14), representing a tetrapolar type. Furthermore, the Pt64 assembly enabled haplotype-based evolutionary analyses for 21 Australian *Pt* isolates, which highlighted the importance of a haplotype resolved reference when inferring genetic relationships using whole genome SNPs. This Pt64 assembly at chromosome-scale with full phase information provides an invaluable resource for genomic and evolutionary research, which will accelerate the understanding of molecular mechanisms underlying *Pt*-wheat interactions and facilitate the development of durable resistance to leaf rust in wheat and sustainable control of rust disease.

## Introduction

The rust fungi are a large group of plant pathogens in the order *Pucciniales* that are damaging in agriculture and in forestry. The rust species *Puccinia triticina* (*Pt*) causes leaf rust on cereals and grasses and is the most commonly occurring cereal rust disease worldwide (Huerta-Espino et al., 2011). *Pt* has also been rated as the most damaging pathogen of wheat, causing global losses of approximately 3.25% as documented by a recent survey of the global burden of pathogens on major food crops (Savary et al., 2019). To prevent the disease, planting wheat with resistance genes (R genes) is both effective and environmentally friendly. However, genetic mutations give rise to new pathotypes that can overcome plant resistance, as exemplified by the observation of *Pt* populations highly diverse for virulence with many different pathotypes detected annually (Park et al., 2000; Aoun et al., 2019). The molecular mechanisms underlying host-pathogen co-evolution include pathogen evasion of host recognition by modifying genes encoding avirulence proteins (Avrs) that are recognized by host proteins encoded by R genes. This specific recognition between host and pathogen was first described by Flor as the gene-for-gene model (Flor, 1971), and this type of host immune response was defined as effector-triggered immunity in differentiation with pathogen-associated molecular pattern-triggered immunity (Jones and Dangl, 2006; Chen et al., 2017).

*Pt* is an obligate biotroph with a complex life cycle consisting of both asexual and sexual stages (Bolton et al., 2008; Cuomo et al., 2017). While the dikaryotic urediniospores (n + n) are most common and can be repeatedly produced through vegetative polycycle on the primary host wheat, the haploid basidiospores (n) generated through meiosis in mature teliospores (from *n* + *n* to 2*n*) have segregated mating-type (MAT) loci (+ and −) and can infect alternate hosts (e.g., *Thalictrum speciosissimum*), where sexual reproduction occurs (Bolton et al., 2008; Cuomo et al., 2017). The subsequently developed pycniospores and receptive hyphae with compatible MATs (opposite MATs) can fuse to restore the dikaryotic state (*n* + *n*). This leads to the production of aeciospores capable of infecting the primary host wheat to once again generate urediniospores. For sexual reproduction in *Pt*, two pairs of MAT genes are required. One pair encodes premating lipopeptide pheromones and their cognate receptors (designated as *a* locus in *Ustilago maydis*), and the other encodes two classes of homeodomain (HD) transcription factors (HD1 and HD2; *b* locus) (Nieuwenhuis et al., 2013). In addition to sexual reproduction, it has been proposed that two distinct compatible MATs are essential for the formation and maintenance of a stable dikaryotic state (Park and Wellings, 2012). It has also been suggested that the maintenance of MAT genes in dikaryotic fungi could have profound implications for their lifestyle, as exemplified by the adaption of Fusarium using pheromone receptors to detect the presence of potential plant hosts (Wallen and Perlin, 2018). Despite their crucial functions, the MAT genes of the wheat rust pathogens have not been investigated thoroughly and much remains to be explored (Cuomo et al., 2017).

For the genomic research on rust fungi, until recently, most published rust genome assemblies are based largely on short-read sequencing, such as the initial assembly of the three wheat rust fungi, *Puccinia graminis* f. sp. *tritici* (*Pgt*), *Puccinia striiformis* f. sp. *tritici* (*Pst*), and *Pt* (Cuomo et al., 2017). Due to the technical limitations of short-read sequencing and the high level of heterozygosity and repetitive sequences in rust genomes, these assemblies are highly fragmented, and haplotype phasing can hardly be achieved (Aime et al., 2017). With the advent of long-read sequencing (LRS), which routinely generates reads longer than 10 kb, more accurate and complete genome assemblies with haplotype phasing have become available (Amarasinghe et al., 2020). For example, several LRS-based genome assemblies have been released for rust fungi, such as those for *Pst* isolates 104E 137 A- and 11-281 (Schwessinger et al., 2018; Li et al., 2019b), the Australian *Pt* isolate Pt104 (Wu et al., 2020), and two isolates of the oat crown rust pathogen *Puccinia coronata* f. sp. *avenae* (Miller et al., 2018). Recently, the LRS-based *Pgt21-0* assembly was combined with Hi-C scaffolding data to yield the first chromosome-scale assembly for *Pgt* (Li et al., 2019a). Characterized by significantly improved contiguity and haplotype phase information, these LRS-based assemblies have provided references with better resolution for comparative studies on Avr genes, structure variations (SVs), and genetic relationships (Li et al., 2020b; Schwessinger et al., 2020; Wu et al., 2020).

In the present study, we used LRS and Hi-C sequencing data to generate a haplotype-resolved assembly at chromosome-scale for *Pt* at the dikaryotic stage using the Australian isolate Pt64 initially detected in 1990 (Park et al., 1999). Comprising 18 chromosome pseudomolecules in each haplotype, this new assembly provided insights into the genetic diversity at SV level between haplotypes of the dikaryotic genome of *Pt*, revealed the complex mating system of *Pt*, and enabled an investigation of evolutionary relationships of 21 Australian *Pt* isolates at the haplotype level based on resequencing data. This study not only provides the complete chromosome-level assembly of *Pt* with haplotype resolution, but also demonstrates the great potential of haplotype-resolved genomic analyses for better understanding of *Pt* biology and evolution.

## Results

### Haplotype-Phased Assembly of *Pt* at Chromosome-Scale

**FIGURE 1:**
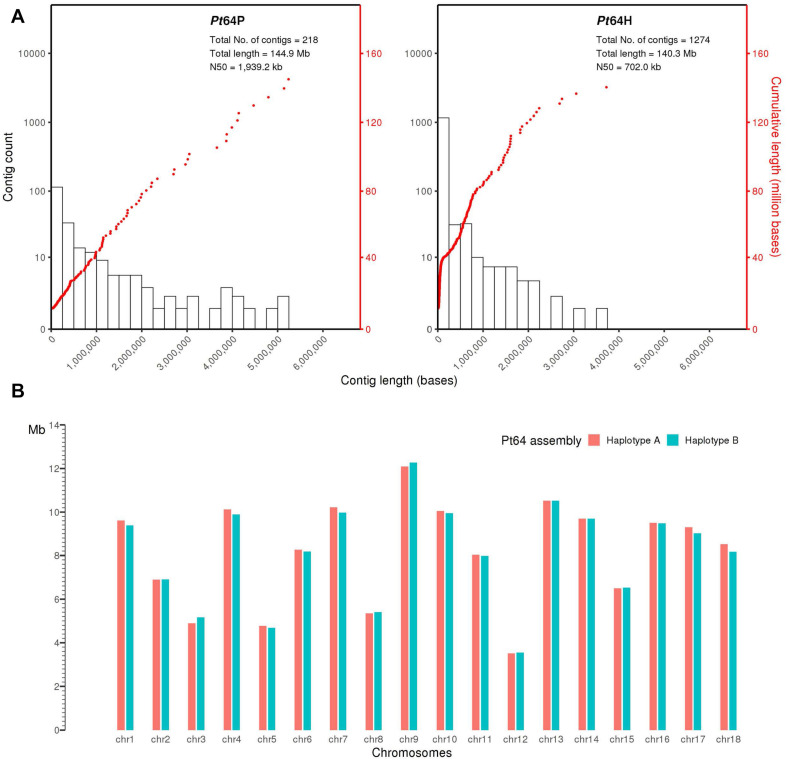
Pt64 assembly at contig and chromosome levels. **(A)** The plot for Pt64 primary contigs (Pt64P) and the associated haplotigs (Pt64H). The log10 counts of contigs within each size bin are shown by histograms with the left *y*-axis. Each dot represents a single contig of a given size corresponding to the *x*-axis. The cumulative sizes of contig lengths sorted from small to large are shown by the dots with the right *y*-axis. **(B)** Schematic representation of the assembled chromosomes for each haplotype of Pt64 assembly.

We generated a complete chromosome-scale assembly of *Pt* using LRS and Hi-C data for the Australian isolate Pt64. A total of 29.8 Gb LRS data with an average read length of 11.3 kb and ∼200-fold coverage were obtained from four SMRT cells using the PacBio Sequel System. Following the integrated Falcon and Falcon-unzip pipeline (Chin et al., 2016), the data were *de novo* assembled which produced a contig assembly consisting of 218 primary contigs (*N*_50_ of 1.9 Mb; total length of 144.9 Mb) with associated haplotigs totaling up to 140.3 Mb (Table 1 and Figure 1). The contig assembly was then integrated with Hi-C data (∼160-fold coverage) for further phasing and scaffolding, which yielded a fully-phased assembly at the chromosome-scale for the dikaryotic genome of *Pt* (referred to as haplotypes/genomes A and B). The haplotypes A and B of Pt64 had a total length of 148.2 and 147.1 Mb and scaffold *N*_50_ of 9.5 and 9.4 Mb, respectively (Table 1). Within each haplotype, 18 chromosome pseudomolecules were constructed, which covered 99% of the total sequence length and ranged from 3.5 to 12.3 Mb in size (Figure 1 and Supplementary File 1). The completeness of the Pt64 assembly was assessed using BUSCO analysis for both haplotypes and revealed similar statistics for each, implicating that each haplotype represented a full haploid genome. Using the A haplotype as the representative (hereafter the A haplotype is used as representative when both haplotypes show similar characteristics), 92.2% of the BUSCO genes were present as complete sequences and 85.8% were in single copy status (Table 1). The fragmented and missing BUSCO genes were 4.3 and 3.5%, respectively. Of the 18 chromosomes in each haplotype of Pt64, 11 telomeres on nine chromosomes of the Pt64 assembly were identified, of which two contained telomere sequences at each end (chromosomes 7 and 11).

**TABLE 1:** Pt64 assembly statistics and completeness evaluation.

Contig assembly statistics		
**Assembly**	**Pt64_primary_contigs**	**Pt64_haplotigs**
Number of contigs	218	1274
Largest contig (bp)	5,240,940	3,723,857
Total length (bp)	144,895,289	140,347,126
GC (%)	46.63	46.67
N50 (bp)	1,939,216	702,014

**Scaffolded assembly statistics**		

**Assembly**	**Pt64_haplotypeA**	**Pt64_haplotypeB**

Number of scaffolds	18	18
Number of unscaffolded contigs	14	14
Largest scaffold (bp)	12,087,149	12,265,344
Total length (bp)	148,180,388	147,118,663
GC (%)	46.64	46.64
N50 (bp)	9,506,076	9,370,800

**Scaffolded assembly BUSCOs**	**Pt64_haplotypeA**	**Pt64_haplotypeB**

Complete BUSCOs (%)	92.2	92.6
Complete and single-copy BUSCOs (%)	85.8	85.8
Complete and duplicated BUSCOs (%)	6.4	6.8
Fragmented BUSCOs (%)	4.3	3.4
Missing BUSCOs (%)	3.5	4.0

### Structural Variations Between Pt64 Haplotypes

**FIGURE 2:**
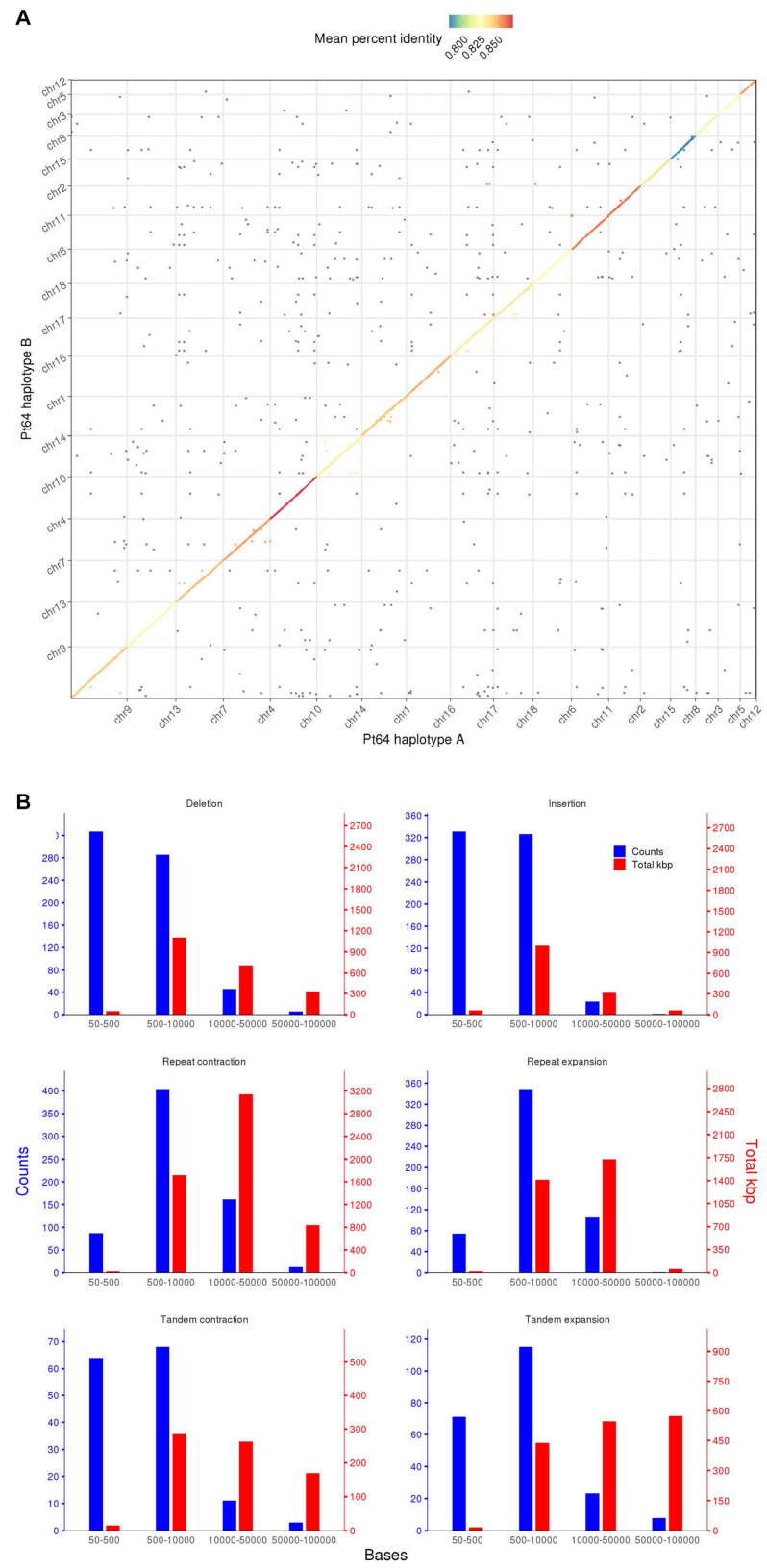
Whole genome alignment and structure variation between haplotypes A and B of the Pt64 assembly. **(A)** Dot plot of sequence alignment of Pt64 chromosome pseudomolecules of haplotypes A and B. **(B)** Summary of structure variation between haplotypes A and B. The counts of structure variation within each size bin are shown by bars with the left *y*-axis, whereas the total bases (kbp) of the structure variation are shown with the right *y*-axis. Each blue and red bar indicates the total counts and the number of bases that are covered in each bin by the specific variation category, respectively.

To assess similarity of the two haplotypes, whole-genome alignments were performed between A and B haplotypes using minimap2 and visualized by dotPlotly (Figure 2) (Li, 2018). The dot plot depicted the collinearity of A and B chromosomes and displayed the average identity of alignment blocks within each pair of homologous chromosomes ranging from 78 to 87% (Figure 2). When the proportion of the total base aligned versus the chromosome length was taken into account, the overall identity between A and B haplotypes was 70–81%.

Using the highly contiguous haplotype assembly, we inspected the SVs between the two haplotypes in Pt64 using Assemblytics as previously described (Nattestad and Schatz, 2016; Schwessinger et al., 2018). Three categories of SVs (50–100 kb) including insertions/deletions, tandem expansions/contractions, and repeat expansions/contractions were identified and 1,343, 363, and 1,193 events were detected for each category, respectively (Figure 2 and Supplementary File 2). While most insertion/deletion and tandem expansion/contraction events were populated in the two bins (50–500 bp and 500–10,000 bp), the repeat expansion/contraction was mainly concentrated in the bin of 500–10,000 bp (Figure 2). When the calculation was based on the length of the affected base pairs, the largest portion of the insertion/deletion and tandem contraction was in the bin of 500–1,000 bp, and the largest portion of repeat expansion/contraction was in the bin of 10–50 kb. Large-scale SVs (50–100 kb) including six insertions/deletions, 13 repeat expansions/contractions, and 11 tandem expansions/contractions were also detected (Figure 2 and Supplementary File 2).

### Genome Annotation and Secretome Prediction

Repeat content including interspersed repeats and non-element repeats was identified using *de novo* predicted repeats and fungal elements from RepBase, which covered 58–59% of the whole genome in each haplotype of Pt64 (Table 2). Despite unclassified repeats, the most abundant repetitive elements were long terminal repeats (16–18%), followed by DNA elements (∼5%).

**TABLE 2:** The repeat contents identified in A and B haplotypes of Pt64 assembly.

Interspersed repeats (%)	Pt64 haplotype A	Pt64 haplotype B
Long interspersed nuclear elements (LINES)	0.62	0.65
Long terminal repeats (LTR) elements	16.47	18.54
DNA elements	5.81	5.15
Unclassified	35.53	34.70
Total interspersed repeats	58.42	59.04
**Non-element repeats (%)**		
Simple repeats	2.74	0.85
Low complexity	0.07	0.07

**FIGURE 3:**
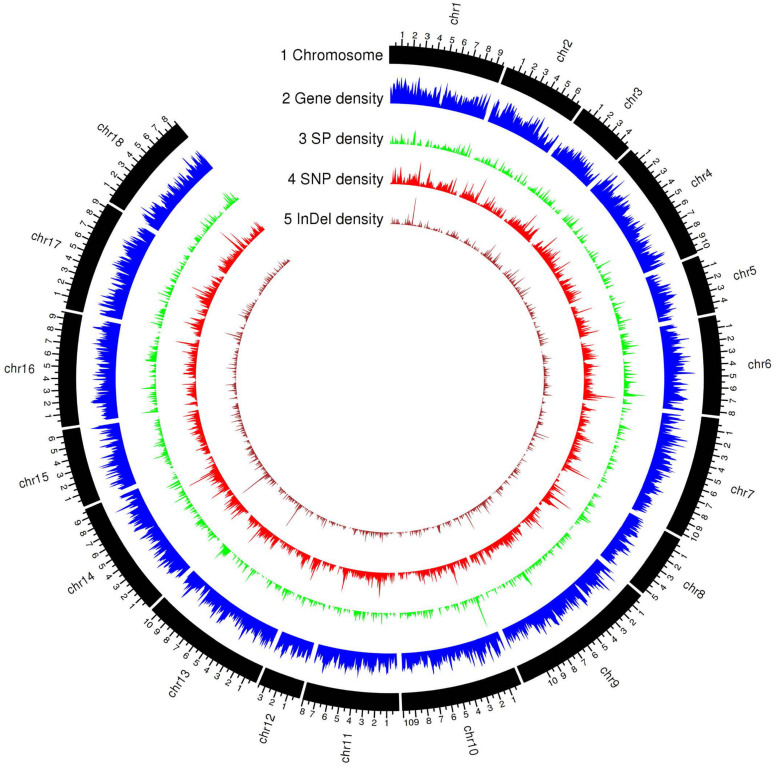
Genomic landscape of predicted gene and secreted protein in the haplotype A of Pt64 assembly and genetic variations of the 21 *Pt* isolates. Tracks from outside to inside are: (1) chromosome name; (2) – (5) gene density, secreted protein density, SNP (single-nucleotide polymorphism), and Indel (insertion or deletion) density in non-overlapping 100 kb windows. Each major tick on the contig track is for 1 Mb length.

The current transcript-based annotation predicted ∼29,000 genes for each haplotype, of which ∼28,000 were protein-encoding genes (Table 3, Figure 3, and Supplementary File 3). The predicted genes were then subjected to functional annotation using curated databases such as CAZymes (carbohydrate active enzymes) and MEROPS (peptidase database), which led to the identification of ∼430 CAZymes in the Pt64 genome with glycoside hydrolase as the most populated subclass and ∼300 proteases with serine peptidase being the most populated family (Table 3).

**TABLE 3:** Gene prediction and functional annotation for haplotypes of Pt64 assembly.

	Pt64 haplotype A	Pt64 haplotype B
**Gene prediction**		
Total number of genes	29,871	29,821
Mean gene length (bp)	1,379	1,399
genome % covered by genes	27.8	28.4
Total number of proteins	28,699	28,656
**Secretome prediction**		
Secreted proteins (SPs)	2,175	1,899
SP % of total proteins	7.6	6.6
**Functional annotation**		
CAZy enzymes total number	435	427
CAZy enzymes GH^*a*^ number	228	224
Proteases total number	306	308
A^*b*^	24	30
C^*b*^	72	68
M^*b*^	71	75
S^*b*^	104	99
T^*b*^	26	27
I^*b*^	9	9

*^*a*^GH, glycoside hydrolase.*

*^*b*^Five classes of peptidases including serine (S), cysteine (C), metallo (M), threonine (T), and aspartic proteases (A) as well as one protease inhibitors class (I).*

For secretome prediction, proteins possessing a signal peptide with no transmembrane segment and a predicted localization of “secreted” or “unknown” were predicted as SPs. In total, 2,175 and 1,899 genes were predicted to encode SPs in the A and B haplotypes of the Pt64 assembly (Table 3, Figure 3, and Supplementary File 4), which comprised about 7.6 and 6.6% of the total proteins of each haplotype, respectively. About 77% of the secretome proteins show no homology to proteins with known functional domains (Supplementary File 4). We examined the expression of these predicted genes in previous *Pt* transcriptomes (Cuomo et al., 2017; Duan et al., 2021) and found that over 20% of these genes encoding the secretome proteins were differentially expressed in infected wheat leaves as compared to urediniospores (Supplementary File 5).

### The Identification of the MAT Genes Located on Two Chromosomes

**FIGURE 4:**
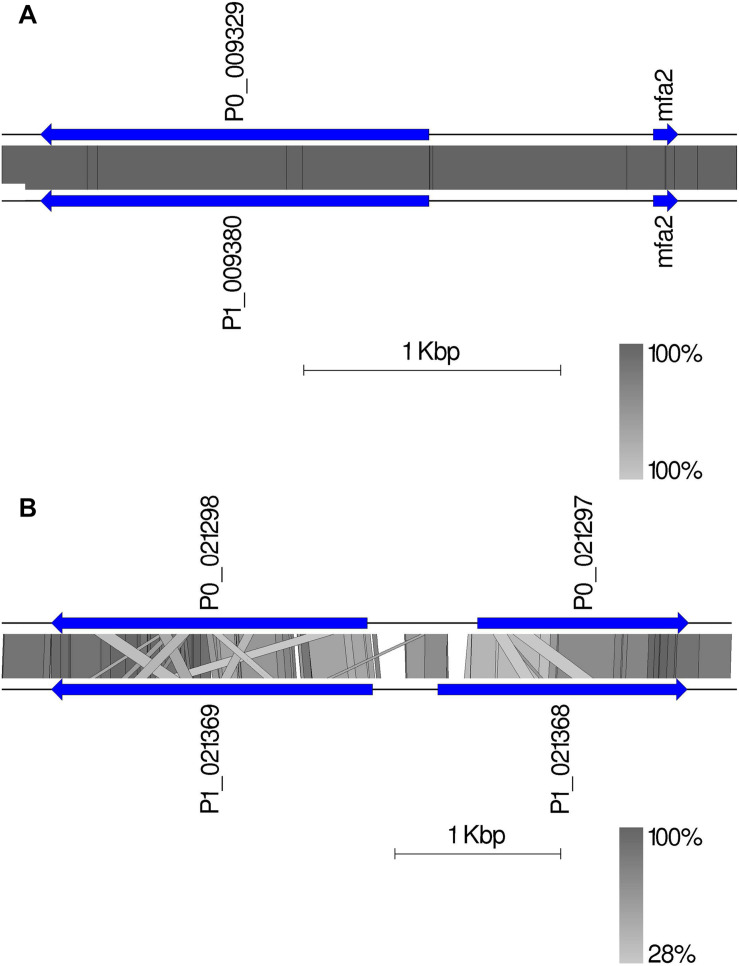
The mating type genes in Pt64. **(A)** The organization and synteny plot of *a* locus including Ptmfa2 and STE3.2 genes (P0_009329 and P1_0099380 correspond to genes GN64P176_009329 and GN64H176_009380, respectively) located on chromosome 7 of A and B haplotypes of Pt64. **(B)** The organization and synteny plot of *b* locus including HD1 (P0_021298 and P1_021369) and HD2 genes (P0_021297 and P1_021368) located on chromosome 14 of A and B haplotypes of Pt64. Genes are labeled with their locus tag and represented by blue rectangle arrows Vertical gray shading illustrates the blastn identity between sequences on both haplotypes, according to the scale shown in the right bottom corner next to the sequence scale bar.

Given the crucial function and lack of understanding of the MAT genes in *Pt*, we investigated them using the annotated haplotypes of Pt64. The Pt64 assembly at chromosome-level clearly showed that the MAT loci *a* (STE3/mfa) and *b* (HD complexes) were independently located on two chromosomes (chromosomes 7 and 14) (Table 4 and Figure 4). For locus *a* on chromosome 7, we observed a pair of genes encoding pheromone (mfa2) and pheromone receptor (STE3.2) with a distance of 874 bp (Table 4 and Figure 4). The variations in the *a* locus were limited to one amino acid difference between STE3.2 genes in the two haplotypes. For locus *b*, we detected a pair of divergently transcribed genes encoding transcription factors bW-HD1 and bE-HD2 on chromosome 14. Compared with the previously described alleles *b1* and *b2* in *Pt* Race 1 (Cuomo et al., 2017), two novel alleles *b3* and *b4* were found in Pt64. For haplotype A, *bW3-HD1* (*P0_021298*) and *bE3-HD2* (*P0_021297*) were 664 bp apart, encoding polypeptides of 612 and 374 amino acids, respectively (Figure 4). For haplotype B, *bW4-HD1* (*P1_021369*) and *bE4-HD2* (*P1_021368*) were 393 bp apart, encoding polypeptides of 621 and 450 amino acids, respectively. Except for bW4-HD1 (P1_021369 in Figure 4) sharing 97% amino acid identity with bW2-HD1 in Race 1, the remaining *b* alleles shared 78–84% amino acid identity, which indicated that the *b* locus of the MAT system in *Pt* is most likely multi-allelic.

**TABLE 4:** Mating type genes in Pt64 assembly.

	Pt64 haplotype A			Pt64 haplotype B			
**Loci a**							

**Gene name**	**Gene ID**	**Coordinates**	**Strand**	**Gene name**	**Gene ID**	**Coordinates**	**Strand**

STE3.2	GN64P176_009329	chr7:830490-832003	−	STE3.2	GN64H176_009380	chr7:830544-832057	−
mfa2	NA^*a*^	chr7:832877-832975	+	mfa2	NA^*a*^	chr7:832931-833029	+

**Loci b**							

**Gene name**	**Gene ID**	**Coordinates**	**Strand**	**Gene name**	**Gene ID**	**Coordinates**	**Strand**

bW3-HD1	GN64P176_021298	chr14:2399576-2401486	+	bW4-HD1	GN64H176_021369	chr14:2400175-2402117	+
bE3-HD2	GN64P176_021297	chr14:2397633-2398912	−	bE4-HD2	GN64H176_021368	chr14:2398274-2399782	−

*^*a*^NA, not applicable.*

### Comparison of Gene Content and Prioritization of *AvrLr20* Candidates

To inspect our annotation results, ortholog analyses were carried out for the two Pt64 haplotypes and the *Pt* Race1 genome, which identified 20,825 orthogroups showing corresponding orthologs between these assemblies (Supplementary File 6). This included 9,716 orthogroups that are specific to the A and B haplotypes of isolate Pt64, and 11,109 orthogroups with at least one ortholog from both isolates. Of the total orthogroups, 19,113 were single-copy orthologs either between the Pt64 haplotypes or across both isolates. Within each genome, genes with at least one ortholog consisted more than 72% of the total protein-encoding genes, reflecting a good consistency in gene annotation across assemblies. For the predicted secretome of haplotypes A and B of Pt64, more than 82% of the SPs had at least one ortholog (Supplementary File 7).

To further examine the assembly, we used 20 candidates of *AvrLr20* previously detected based on the Race 1 genome (Wu et al., 2017) in the context of the phased orthologs in Pt64. Based on our phenotype studies and previous genetic analyses (Park et al., 1999), we postulated that Pt64 most likely possesses two heterozygous alleles with one copy avirulent and the other virulent to *Lr20*. If this assumption holds, the ortholog status could be transformed into an additional criterion that at least one copy of the *AvrLr20* candidate ortholog shall be present in the Pt64 assembly as Pt64 is avirulent to *Lr20* and the two orthologs of the *AvrLr20* candidate in Pt64 shall be heterozygous. Of the 20 candidates showing no known functional domains (Supplementary File 4), 13 had at least one ortholog with Pt64, and nine were heterozygous (Table 5). Most of these heterozygous candidates were not differentially regulated *in planta* as compared to that in urediniospores based on the previous *Pt* transcriptomes (Cuomo et al., 2017; Duan et al., 2021) except for two, GN64P176_026812/GN64H176_026857 and GN64P176_007373 (Table 5). Detailed functions of these nine candidates will be further prioritized and validated in future studies.

**TABLE 5:** The Pt64 orthologs corresponding to the *avrLr20* candidates derived from Race 1 reference genome.

Candidate name	Gene.size	Ortho_pt64HapA^*a*^	*In planta* differential expression^*d*^	Ortho_pt64HapB^*b*^	*In planta* differential expression^*d*^	AB_heterozygous^*c*^
PTTG_25257	125	GN64P176_013699	No	GN64H176_013750	No	Yes
PTTG_25496	180	GN64P176_026812	Yes	GN64H176_026857	Yes	Yes
PTTG_06625	504	GN64P176_006082	No	GN64H176_006162	No	Yes
PTTG_00930	196	GN64P176_022159	No	GN64H176_022235	No	No
PTTG_26540	520	GN64P176_000705	No	GN64H176_000708	No	Yes
PTTG_03866	499	GN64P176_007898	No	GN64H176_007939	No	No
PTTG_06324	296	GN64P176_016636	No	GN64H176_016618	No	Yes
PTTG_06325	280	GN64P176_016638	No	GN64H176_016620	No	Yes
PTTG_03715	453	GN64P176_021245	No	GN64H176_021313	No	No
PTTG_08794	134	GN64P176_003576	No	GN64H176_003560	No	No
PTTG_29551	114	GN64P176_007378	No	GN64H176_007444	No	Yes
PTTG_09239	190	GN64P176_007373	Yes	GN64H176_007443	No	Yes
PTTG_29866	805	GN64P176_003999	No	GN64H176_004046	No	Yes

*^*a*^Ortholog in haplotype A of Pt64.*

*^*b*^Ortholog in haplotype B of Pt64.*

*^*c*^The heterozygous status of the orthologs in the dikaryotic genome of Pt64.*

*^*d*^Differential expression analyzed based on RNA-seq data from Cuomo et al. (2017) and Duan et al. (2021).*

### Whole-Genome Sequencing Analysis Based on Pt64 Assembly

To evaluate the Pt64 assembly, we used it to investigate genetic variants and relationships within a set of Australian *Pt* isolates including the Illumina sequencing data of Pt64 and 20 *Pt* isolates that are publicly available (Wu et al., 2017). For haplotype A, the Pt64 reference genome was covered by between 97.4 and 99.2% of the total genome bases (Supplementary File 8). The average aligned read depth was 28.4 and the average mapping rate of these isolates was 90.9%, which was a substantial improvement over the previous mapping rates of 74–81% using the *Pt* Race 1 reference genome (Cuomo et al., 2017) (Supplementary File 8). For each isolate, genome-wide polymorphisms were detected using GATK HaplotypeCaller based on reads mapped to the Pt64 genome (Figure 3). The average number of total variants identified was 548,032 and the average number of single nucleotide polymorphism (SNP) and insertion-deletion mutation (InDel) variants were 476,509 and 58,222, respectively (Supplementary File 9). The average rates of heterozygous variants (SNP and InDel) and SNPs were 2.9 variants/kb and 2.7 SNPs/kb, respectively. The mapping and variant statistics derived from haplotype B were similar to those from haplotype A (Supplementary Files 8, 9) and both were also in line with reports from previous whole-genome sequencing studies on *Pt* (Cuomo et al., 2017; Wu et al., 2017, 2020).

### Phylogenetic Analysis of 21 Australian *Pt* Isolates

**FIGURE 5:**
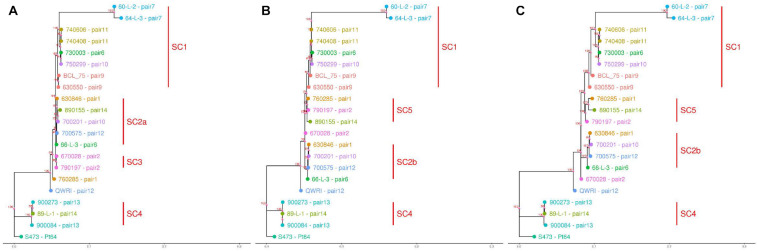
Dendrograms of 21 *Pt* pathotypes inferred from whole-genome SNPs and individual haplotype genomes. The bottom bar indicates number of nucleotide substitutions per site. The numbers shown on the dendrogram branches are the percentage of bootstrap replicates (1,000) supporting the cluster. Panels **(A–C)** are dendrograms inferred using haplotype A of Pt64, haplotype B of Pt64, and whole-genome SNPs detected against the full dikaryotic genome of Pt64, respectively.

Based on the SNPs identified, phylogenetic analysis was carried out using the full dikaryotic genome of Pt64 and the individual haplotype genome of Pt64 separately. Using whole-genome SNPs called against the full dikaryotic genome of Pt64, the phylogenetic tree derived from maximum likelihood method showed similar overall topology to those derived from filtered SNPs called against A or B haplotype (Figure 5). All three phylogenies had three major branches, which linked to Pt64 (S473), the isolates in subclade 4 (SC4), and the remaining 17 isolates. All three isolates linked to SC4 (Figure 5) were collected around 1990 and identified as International Race 104; for the remaining 17 isolates linked to the third major branch, the two isolates from pair 7 forming a sister group showed larger distance than the others. It was noted that the third major branch inferred from the full dikaryotic genome (Figure 5C) was longer than those inferred from Pt64 haplotypes (Figures 5A,B), indicating that the estimated genetic distance for this subclade was larger based on the full genome of Pt64 than that based on a single haplotype. In addition, some pronounced differences were observed for the phylogenetic tree using filtered SNPs called against haplotype A versus that against haplotype B. For example, in the phylogeny inferred from A genome, pair 2 isolates (670028 and 790197; SC3) formed a sister group (Figure 5A), whereas in the other two phylogenies, the two isolates were separated (Figures 5B,C). Similarly, we also observed the relocation of the pair 1 isolate 760285 from the position close to SC3 to SC5 and the relocation of the pair 14 isolate (890155) from SC2a to SC5 when the B genome was used as the reference (Figures 5B,C).

## Discussion

Despite the economic importance of *Pt* worldwide, genomic resources for this pathogen are limited. Currently, no chromosome-level phased assemblies of *Pt* are available, which has greatly hampered detailed investigations of genetic variation, evolutionary relationships with haplotype resolution, and Avr gene cloning. Built upon the recent technology breakthroughs of LRS and Hi-C sequencing, here we present a phased chromosome-scale genome assembly of *Pt* using the Australian *Pt* pathotype, Pt64. Sharing the same number of chromosomes observed in several closely related rust fungi such as *Pgt* and *Melampsora lini* (*M. lini*) (Boehm and Bushnell, 1992; Li et al., 2019a), each haplotype of the dikaryotic genome of Pt64 has 18 chromosome pseudomolecules that range in size from 3.5 to 12.3 Mb (Table 1 and Figure 1). The Pt64 assembly at chromosome-scale with two highly contiguous haplotypes provides an unprecedented opportunity to investigate haplotype diversity at the SV level, revealing variations previously undetected by short-read based assemblies. Using this chromosome-scale assembly, we explored haplotype diversity at SV level and the complex mating system of *Pt*. This assembly has also enabled us to analyze the Illumina sequencing data of 21 Australian *Pt* isolates to explore the genetic relationships based on individual haplotype genomes, which has never been attempted for *Pt* before.

With the fully-phased Pt64 assembly at chromosome-level providing a valuable platform for SV investigation, 10% of the total genome was found to be represented by SVs between haplotypes A and B of Pt64 (Figure 2 and Supplementary File 2), highlighting a hidden layer of inter-haplotype variation previously undetected by short-read based assemblies. The 10% SV in *Pt* is slightly higher than the 8.6% SV reported for *Pgt* (Li et al., 2019a), which is in line with the observation of genome expansion by the integration of repetitive elements in *Pt* as compared to *Pgt* suggested by a previous study by Cuomo et al. (2017). In the *Pst* isolate Pst-104E, SVs comprised 6.4% of the total size of the primary assembly and the actual level of SVs was postulated to be higher (Schwessinger et al., 2018). Further to these findings that SVs do represent a considerable portion of the wheat rust genomes, the importance of SVs in the pathogenicity of rust fungi was also highlighted by our recent identification of *AvrSr50*, which unveiled a ∼200 bp insertion in the *AvrSr50* gene leading to the development of virulence (Chen et al., 2017). It is thus anticipated that with Pt64 assembly facilitating more accurate and in-depth detection of SVs along with the development of new approaches integrating SV analysis in rust comparative genomics (Song et al., 2020), our understanding of the impact of SVs on rust pathogenicity will be substantially accelerated.

The annotation of the newly built Pt64 assembly (Tables 2, 3 and Figure 3) enabled the detection and characterization of the MAT genes, showing that the two MAT loci *a* (P/R genes) and *b* (HD genes) occur on two separate chromosomes (chromosomes 7 and 14) and implicating a tetrapolar type of mating system in *Pt* (Table 4 and Figure 4). For many species in the phylum Basidiomycota, the two sets of MAT genes that control different stages of the sexual cycle govern sexual reproduction, which not only promotes genetic variation essential for adaptation and long-term survival, but also plays a central role in pathogenic development (Maia et al., 2015). While early studies reported the tetrapolar type of mating system in the rust species *P. coronata*, *M. lini*, and *Cronartium quercuum* (Kües et al., 2011), the most recent genome sequencing of *Pgt* revealed that the MAT system of *Pgt* consists of single independent di-allelic (*a*) and multi-allelic (*b*) loci (Li et al., 2019a). In the current Pt64 assembly, the STE3.2 allele in haplotype B was identical to that reported in *Pt* Race 1 (Cuomo et al., 2017), indicating that the *a* locus of *Pt* could be di-allelic. A similar configuration was also observed in most of the known tetrapolar yeast species such as *U. maydis* and *Cryptococcus amylolentus* (Bölker et al., 1992; Kämper et al., 1995; Findley et al., 2012). Previously, it was proposed that the bipolar states in basidiomycetes have most likely arisen from a tetrapolar configuration. Given that the maintenance of MAT genes may have more profound meaning for the adapted lifestyle of fungi as suggested by a recent study (Wallen and Perlin, 2018), it is plausible to postulate that the emergence of bipolar states could be a strategy to balance between the requirement for maintaining MAT genes and the cost of keeping such a complex system.

We attempted an ortholog analysis of the two haplotypes of Pt64 to prioritize previously identified candidates for *AvrLr20* by inspecting the heterozygous status of the candidate orthologs in Pt64 (Table 5). In our previous study, we integrated genome-wide association with comparative analysis to obtain a panel of *AvrLr20* candidates based on the *Pt* Race 1 reference (Wu et al., 2017). The haplotype-resolved Pt64 assembly has provided a new opportunity to further prioritize these candidates by inspecting the heterozygous status of each candidate gene in Pt64, which further prioritized nine candidates. While additional criteria such as *in planta* expression and similarity to haustorial proteins have been used to overcome the difficulty in prioritizing candidate Avr genes by previous studies (Prasad et al., 2019), we have explored a new scenario integrating heterozygous status postulation from phenotype studies (Park and Wellings, 2012) with ortholog and haplotype information of phased *Pt* assembly to prioritize Avr candidate genes. In aid of the high quality of Pt64 assembly, this approach has demonstrated the potential value of specific phenotypic information from rust survey analyses when applied in conjunction with genomic resources of haplotype and ortholog information for functional genomics of rust fungi.

The phased genome of Pt64 allowed us to perform haplotype-based phylogenetic analyses (Figure 5), which demonstrated some pronounced differences between phylogenies of 21 *Pt* isolates derived from the three reference genomes, namely haplotype A, haplotype B, and the full genome (A and B), despite the overall similarity. This contrast suggests that the filtered SNPs derived from genome A may place more emphasis on the similarity between pair 2 isolates, whereas the SNPs based on genome B or the entire genome of A and B may place more emphasis on the differences between these two isolates. Despite the similar overall topology, the aforementioned incongruities between phylogenetic trees reveal the potential complexity hidden behind inferred genetic relationships based on whole-genome SNP data without haplotype resolution and the potential limitations of using a single haplotype as the reference to fully reveal genetic relationships. Consistent with our observation, a previous *Pgt* study also reported that Ug99 and five international isolates were relocated to different subclades when different haplotypes of the reference genomes were used (Li et al., 2019a). Taken together, both studies clearly demonstrate the hidden complexity in deriving genetic relationships using whole genome SNPs called against a reference without haplotype resolution. Similarly, the use of different reference genomes may also impact other outcomes of comparative analyses based on sequence alignment and SNP calling such as the identification of Avr genes. Most sequence data analyses have used the conventional comparative approach, which starts by aligning sequence reads to a linear reference genome. Because this does not take into account the multiple genomes at haplotype level, a new approach effectively using these haplotype genomes is needed. Recently, a new strategy known as reference flow has been proposed, which can simultaneously use the information from multiple reference genomes to improve alignment accuracy and reduce reference bias (Chen et al., 2020). In addition to the new alignment strategy, building pan-genomes is also a well-documented strategy (Tettelin et al., 2005; Golicz et al., 2020; Liu et al., 2020). While a single linear reference genome cannot fully represent the genetic diversity of a species, a pan-genome consisting of both core genes/sequences (present in all individuals) and variable genes/sequences (present in some individuals) can (Hurgobin and Edwards, 2017; Bayer et al., 2020). With this full representation, a pan-genome can provide more accurate and comprehensive information critical for comparative genomics such as analyses for phylogeny and identification of Avr genes.

With the advent of LRS technologies, more and more higher quality assemblies with significantly improved contiguity have become available, as exemplified by the Pt64 assembly presented here and recently available assemblies such as Pt104, Pgt21-0, and Pst104E (Schwessinger et al., 2018; Li et al., 2019a; Wu et al., 2020). For these high-quality assemblies, various new methods have been proposed for pan-genome construction. For example, a graph-based data model is suggested for the construction of a pan-genome graph, which by preserving links and relationships between pan-genome sequences, can compactly encode tens of thousands of SVs previously missing from the traditional lineage reference (Golicz et al., 2020; Li et al., 2020a). Currently, our lab is building high-quality *Pt* assemblies for representative isolates from different lineages collected in Australia, and the construction of a pan-genome capturing the entire gene/sequence space of *Pt* would be the ultimate goal.

In summary, we presented a phased chromosome-scale genome assembly using an Australian *Pt* isolate Pt64, which represents an unparalleled resource for understanding *Pt* diversity, pathogenicity, and evolution. This new assembly has enabled us to compare SVs between haplotypes, to unveil the complex mating system in *Pt*, and to investigate the evolutionary relationships with haplotype resolution for a set of 21 Australian *Pt* isolates. This Pt64 assembly at chromosome-scale with full phase information will undoubtedly accelerate the dissection of the genetic basis and understanding of molecular mechanisms underlying *Pt*-wheat interactions, which will facilitate future efforts to achieve sustainable control of the rust diseases of plants.

## Materials and Methods

### *Pt* Isolates and Plant Inoculation

We used *Pt* isolates identified in nationwide race surveys of Australian cereal rust control program for this study. These isolates are curated in the Plant Breeding Institute Rust Collection, The University of Sydney, Australia. To ensure the purity of each isolate for sequencing, a single pustule was selected from a region of low-density infection and propagated on the wheat genotype Morocco prior to DNA preparation. The identity and purity of each isolate were checked by pathogenicity tests with a set of host differentials at each cycle of inoculum increase and also using urediniospores subsampled from those used for DNA extraction. For *Pt* infection, plants were grown at high density (∼25 seeds per 12 cm pot with compost as growth media) to the one leaf stage (∼7 days) in a greenhouse microclimate set at 18–25°C temperature and with natural day light. Plants were inoculated as previously described (Wu et al., 2017) and mature spores were collected, dried and stored at −80°C for DNA isolation. Pathotype 64-(6),(7),(10),11 (North American race LBBQB; Kolmer and Hughes, 2016) was chosen to develop a high quality assembly based on its avirulence for many of the cataloged leaf rust resistance genes in wheat [*viz*. avirulence/virulence (partial virulence): *Lr2a*, *Lr2b*, *Lr2c*, *Lr3a*, *Lr3bg*, *Lr3ka*, *Lr9*, *Lr11*, *Lr14a*, *Lr15*, *Lr17b*, *Lr18*, *Lr19*, *Lr20*, *Lr21*, *Lr23*, *Lr24*, *Lr25*, *Lr26*, *Lr28*, *Lr29*, *Lr30*, *Lr32*, *Lr37*/*Lr1*, *Lr10*, (*Lr13*), *Lr16*, (*Lr17a*), (*Lr27*+*31*); Park et al., 1999].

### DNA Extraction and Sequencing

For PacBio sequencing, DNA was extracted from urediniospores as previously described (Schwessinger and Rathjen, 2017) and sequencing was performed at the Australian Genome Research Facility Ltd (Adelaide, Australia). For library preparation, the SMRT cell Template Prep Kit 1.0-SPv3 with BluePippin size-selection with 15–20 kb cutoff (PacBio) was used and DNA libraries were sequenced on a PacBio Sequel System with Sequel Sequencing chemistry 2.1. Four SMRT cells were used for Pt64 and each SMRT cell had a 5–10 Gb capacity. Hi-C library preparation and sequencing was carried out by Phase Genomics (Seattle, WA, United States). The Hi-C library of Pt64 was constructed with the ProxiMeta Hi-C kit from Phase Genomics following the standard protocol using the enzyme Sau3A for digestion. For resequencing, TruSeq library of DNA samples for Pt64 was constructed and sequenced on the Illumina HiSeqX instrument at Novogene with as 150 bp paired-end reads (Hong Kong, China).

### Genome Assembly and Scaffolding

The integrated pipeline of FALCON and FALCON-Unzip (v4.1.0) was used for genome assembly (Chin et al., 2016). Read length cutoffs were computed by FALCON based on the seed coverage and expected genome size. After assembly by Falcon, FALCON-Unzip was used to phase haplotypes and to generate consensus sequences for primary contigs and the associated haplotigs. The generated assembly was subjected to error correction using the final consensus-calling algorithm Quiver implemented in SMRT (v4.0.0), an algorithm for calling highly accurate consensus from PacBio reads using a hidden Markov model exploiting both the base calls and QV metrics to infer the true underlying DNA sequence (Chin et al., 2013). Blastn searches against the NCBI nucleotide reference database were used to check potential noneukaryotic contamination and none of the contigs were found to have predominant noneukaryotic sequences as best BLAST hits at any given position.

A Hi-C-based phasing was processed by FALCON-Phase to correct likely phase switching errors in the primary contigs and alternate haplotigs generated from FALCON-Unzip, which created one complete set of contigs for each phase (Kronenberg et al., 2019). This assembly was further processed by the Proximo Hi-C genome scaffolding platform by Phase Genomics (Seattle, WA, United States) to build chromosome-scale scaffolds using the same single-phase scaffolding procedure as previously described (Bickhart et al., 2017). Approximately 40,000 separate Proximo runs were performed to optimize the number of scaffolds and scaffold construction in order to make the scaffolds as concordant with the observed Hi-C data as possible. Juicebox was then used to correct scaffolding errors and FALCON-Phase was run a second time to detect and correct phase switching errors, which produced the final Pt64 assembly, representing a dikaryotic genome with fully phased information for each set of chromosome-scale scaffolds (Durand et al., 2016; Kronenberg et al., 2019). To evaluate the completeness of the final assembly, the software BUSCO (v3.0) (Simao et al., 2015) was used for comparison with the fungal lineage set of orthologs (basidiomycota_odb9), which consisted of 1,335 conserved orthologs of basidiomycete fungi. To identify telomeric sequences in each haplotype of Pt64, 5 kb sequences from the termini of each chromosome were first extracted for repeat identification by tandem repeats finder (TRF) (Benson, 1999), which were then screened for telomeric sequences as described previously (Li et al., 2019a).

### RNA Sequencing, Genome Annotation, and Secretome Prediction

Infected leaves were collected at 3, 5, and 7 days after inoculation with Pt64 and immediately frozen in liquid nitrogen. RNA samples were prepared as previously described (Wu et al., 2020). For library preparation, around 10 μg of total RNA was processed with the mRNA-Seq Sample Preparation kit (Illumina), which was then sequenced on the Illumina HiSeq2500 platform (125 bp paired-end reads).

After trimming, RNA-seq reads were first aligned to the Pt64 genome by using the CLC module large gap read mapping (default parameters). Both *de novo* transcriptome assembly and genome-guided transcriptome assembly using Trinity (v2.1.1) were built (Haas et al., 2013). The transcript models and previously reported EST sequences (Xu et al., 2011) were combined as transcript evidence; *Pt* Race 1 protein sequences were used as protein evidence. This evidence was fed into the Funannotate pipeline (v0.7.2) for a comprehensive annotation of the Pt64 assembly. Funannotate has integrated multiple tools tailored for fungal genome annotation, including repeat identification, alignment of protein and transcript evidence, *ab initio* gene prediction, tRNAs prediction, evidence weighting and combining, and final clean of gene models (Slater and Birney, 2005; Wu and Watanabe, 2005; Haas et al., 2008; Hoff et al., 2016; Lowe and Chan, 2016; Wu et al., 2020). After genome annotation, orthologs between Pt64 and *Pt* Race 1 genomes were identified by Proteinortho v5.16 (synteny mode) (Lechner et al., 2011). Functional annotation to the protein-coding genes was carried out using curated databases including UniProt (Apweiler et al., 2004), Pfam domains (Finn et al., 2014), CAZymes (Yin et al., 2012), and MEROPS (Rawlings et al., 2016). The mating type genes were identified by BLAST searches with the pheromone peptide encoding genes (mfa2 and mfa3) and pheromone receptors (STE3.2 and STE3.3) from the *a* locus, and HD1 and HD2 genes from the *b* locus previously reported in *Pt* Race 1 (Cuomo et al., 2017).

Proteins predicted to have a signal peptide with no transmembrane segment and a TargetP predicted localization of “secreted” or “unknown” were identified as effector candidates. SignalP v4.1 (Dyrløv Bendtsen et al., 2004), TMHMM v2.0 (Krogh et al., 2001), and TargetP v1.1 (Emanuelsson et al., 2000) were used for the prediction of signal peptide, transmembrane domain, and subcellular location, respectively.

For transcriptome analyses, raw sequencing reads (Cuomo et al., 2017; Duan et al., 2021) were trimmed by fastp v0.19.6 (Chen et al., 2018), aligned to each of the Pt64 halotype with HISAT2 v2.2.1 (Pertea et al., 2016). Unique transcripts were assembled by StringTie v1.3.3 (Pertea et al., 2016) which was followed by gene expression analysis using the Bioconductor package DESeq2 (Love et al., 2014) in R.

### Comparative Genomic and Phylogenetic Analyses

The 20 *Pt* isolates cover a range of pathotypes and comprise 10 pairs with the members in each pair contrasting in virulence profile to *Lr20* (Park et al., 1999; Wu et al., 2017). Full virulence/avirulence attributes of these isolates were provided by Wu et al. (2017) and are reproduced here in Supplementary File 8. After trimming, the sequence data as paired-end reads were mapped to the two haplotypes of Pt64 individually. Paired-end Illumina reads of the *Pt* isolates were independently mapped to the reference genome using BWA mem v0.7.17 (Li and Durbin, 2009). High quality alignments (with the mapping quality cutoff of 30) were selected using the SAMTools v1.6 view command and the generated BAM files were used as input to call SNPs and InDels using GATK v4.1.4.1 HaplotypeCaller (McKenna et al., 2010). Based on the genome-wide SNPs identified, the evolutionary relationships of the isolates were inferred using SNPhylo with the performance of 1,000 bootstrap replicates and visualized by the Bioconductor package ggtree v2.0.4 (Lee et al., 2014; Yu et al., 2018). SNPs called against the A or B haplotype were filtered from the total SNP sets using the Bioconductor package GenomicRanges v1.40 (Lawrence et al., 2013). The identified SNPs and InDels were visualized by the R package circlize v0.4.8 (Krzywinski et al., 2009). To obtain a summary of sequence alignments between haplotypes A and B, whole genome alignments were performed with minimap2 (Li, 2018) and visualized with dotPlotly^1^. For the identification of SVs between the two haplotypes of Pt64, mummer3 (Kurtz et al., 2004) was used to align the two haplotypes and the output delta file was fed into Assemblytics (Nattestad and Schatz, 2016) for SV detection.

## Data Availability Statement

The datasets generated for this study can be found in online repositories. The names of the repository/repositories and accession number(s) can be found below: https://www.ncbi.nlm.nih.gov/search/all/?term=PRJNA728188, PRJNA728188.

## Author Contributions

JW analyzed the data and wrote the manuscript. CD, MH, and YD performed the experiments. LS and YD contributed to data analysis and prepared the figures. RP identified all pathotypes used. CD, MH, LS, YD, and RP contributed to the manuscript. RP and JW designed the experiments. RP supervised the work. All authors read and approved the final manuscript.

## Conflict of Interest

The authors declare that the research was conducted in the absence of any commercial or financial relationships that could be construed as a potential conflict of interest.

## Publisher’s Note

All claims expressed in this article are solely those of the authors and do not necessarily represent those of their affiliated organizations, or those of the publisher, the editors and the reviewers. Any product that may be evaluated in this article, or claim that may be made by its manufacturer, is not guaranteed or endorsed by the publisher.

## Footnotes

1
https://github.com/tpoorten/dotPlotly/

## Supplementary Material

Supplementary File 1 Chromosomes sizes in Pt64.

Supplementary File 2 Counts and base pair length of the structure variants between A and B haplotypes of Pt64.

Supplementary File 3 Annotation table for each haplotype of Pt64 assembly.

Supplementary File 4 Annotation of predicted effectors for each haplotype of Pt64 assembly.

Supplementary File 5 Expression of predicted effectors in spores and infected wheat leaves of two *Pt* isolates.

Supplementary File 6 The orthologs between Pt64 A haplotype, B haplotype, and *Pt* Race 1.

Supplementary File 7 Orthologs identified for SP genes of Pt64 haplotypes A and B.

Supplementary File 8 Passport data and mapping information for the 21 Australian *Pt* isolates.

Supplementary File 9 Statistics of the genomic variants in the 21 Australian *Pt* isolates.

## References

[B1] Aime C. M. McTaggart A. R. Mondo S. J. Duplessis S. (2017). Phylogenetics and phylogenomics of rust fungi. *Advanced Genetics* 100 267–307. 10.1016/bs.adgen.2017.09.011 29153402

[B2] Amarasinghe S. L. Su S. Dong X. Zappia L. Ritchie M. E. Gouil Q. (2020). Opportunities and challenges in long-read sequencing data analysis. *Genome Biol.* 21:30. 10.1186/s13059-020-1935-5 PMC7006217 32033565

[B3] Aoun M. Kolmer J. A. Breiland M. Richards J. Brueggeman R. S. Szabo L. J. (2019). Genotyping-by-sequencing for the study of genetic diversity in *Puccinia triticina*. *Plant Dis.* 104 752–760. 10.1094/pdis-09-19-1890-re 31910116

[B4] Apweiler R. Bairoch A. Wu C. H. Barker W. C. Boeckmann B. Ferro S. (2004). UniProt: the universal protein knowledgebase. *Nucleic Acids Res.* 32 D115–D119. 14681372 10.1093/nar/gkh131 PMC308865

[B5] Bayer P. E. Golicz A. A. Scheben A. Batley J. Edwards D. (2020). Plant pan-genomes are the new reference. *Nat. Plants* 6 914–920. 10.1038/s41477-020-0733-0 32690893

[B6] Benson G. (1999). Tandem repeats finder: a program to analyze DNA sequences. *Nucleic Acids Res.* 27 573–580. 10.1093/nar/27.2.573 9862982 PMC148217

[B7] Bickhart D. M. Rosen B. D. Koren S. Sayre B. L. Hastie A. R. Chan S. (2017). Single-molecule sequencing and chromatin conformation capture enable de novo reference assembly of the domestic goat genome. *Nat/Genet.* 49 643–650. 10.1038/ng.3802 28263316 PMC5909822

[B8] Boehm E. W. A. Bushnell W. R. (1992). An ultrastructural pachytene karyotype for Melampsora lini. *Phytopathology* 82 1212–1218. 10.1094/phyto-82-1212

[B9] Bölker M. Urban M. Kahmann R. (1992). The a mating type locus of U. maydis specifies cell signaling components. *Cell* 68 441–450. 10.1016/0092-8674(92)90182-c 1310895

[B10] Bolton M. D. Kolmer J. A. Garvin D. F. (2008). Wheat leaf rust caused by *Puccinia triticina*. *Mol. Plant Pathol.* 9 563–575. 19018988 10.1111/j.1364-3703.2008.00487.x PMC6640346

[B11] Chen J. Upadhyaya N. M. Ortiz D. Sperschneider J. Li F. Bouton C. (2017). Loss of AvrSr50 by somatic exchange in stem rust leads to virulence for Sr50 resistance in wheat. *Science* 358 1607–1610. 10.1126/science.aao4810 29269475

[B12] Chen N.-C. Solomon B. Mun T. Iyer S. Langmead B. (2020). Reducing reference bias using multiple population reference genomes. *bioRxiv [Preprint]* 10.1101/2020.03.03.975219 PMC7780692 33397413

[B13] Chen S. Zhou Y. Chen Y. Gu J. (2018). fastp: an ultra-fast all-in-one FASTQ preprocessor. *Bioinformatics* 34 i884–i890. 30423086 10.1093/bioinformatics/bty560 PMC6129281

[B14] Chin C.-S. Alexander D. H. Marks P. Klammer A. A. Drake J. Heiner C. (2013). Nonhybrid, finished microbial genome assemblies from long-read SMRT sequencing data. *Nat. Methods* 10 563–569. 10.1038/nmeth.2474 23644548

[B15] Chin C. S. Peluso P. Sedlazeck F. J. Nattestad M. Concepcion G. T. Clum A. (2016). Phased diploid genome assembly with single-molecule real-time sequencing. *Nat. Methods* 13 1050–1054. 10.1038/nmeth.4035 27749838 PMC5503144

[B16] Cuomo C. A. Bakkeren G. Khalil H. B. Panwar V. Joly D. Linning R. (2017). Comparative analysis highlights variable genome content of wheat rusts and divergence of the mating loci. *G3 (Bethesda)* 7 361–376. 10.1534/g3.116.032797 27913634 PMC5295586

[B17] Duan H. Jones A. W. Hewitt T. Mackenzie A. Hu Y. Sharp A. (2021). Identification and correction of phase switches with Hi-C data in the Nanopore and HiFi chromosome-scale assemblies of the dikaryotic leaf rust fungus *Puccinia triticina*. *bioRxiv [Preprint]* 10.1101/2021.04.28.441890

[B18] Durand N. Robinson J. Shamim S. Machol I. Mesirov J. p. Lander E. (2016). Juicebox provides a visualization system for Hi-C contact maps with unlimited zoom. *Cell Syst.* 3 99–101. 10.1016/j.cels.2015.07.012 27467250 PMC5596920

[B19] Dyrløv Bendtsen J. Nielsen H. Von Heijne G. Brunak S. (2004). Improved prediction of signal peptides: signalP 3.0. *J. Mol. Biol.* 340 783–795. 10.1016/j.jmb.2004.05.028 15223320

[B20] Emanuelsson O. Nielsen H. Brunak S. Von Heijne G. (2000). Predicting subcellular localization of proteins based on their N-terminal amino acid sequence. *J. Mol. Biol.* 300 1005–1016. 10.1006/jmbi.2000.3903 10891285

[B21] Findley K. Sun S. Fraser J. A. Hsueh Y. P. Averette A. F. Li W. (2012). Discovery of a modified tetrapolar sexual cycle in Cryptococcus amylolentus and the evolution of MAT in the Cryptococcus species complex. *PLoS Genet.* 8:e1002528. 10.1371/journal.pgen.1002528 22359516 PMC3280970

[B22] Finn R. D. Bateman A. Clements J. Coggill P. Eberhardt R. Y. Eddy S. R. (2014). Pfam: the protein families database. *Nucleic Acids Res.* 42 D222–D230. 24288371 10.1093/nar/gkt1223 PMC3965110

[B23] Flor H. H. (1971). Current status of the gene-for-gene concept. *Annu. Rev. Phytopathol.* 9 275–296. 10.1146/annurev.py.09.090171.001423

[B24] Golicz A. A. Bayer P. E. Bhalla P. L. Batley J. Edwards D. (2020). Pangenomics comes of age: from bacteria to plant and animal applications. *Trends Genet.* 36 132–145. 10.1016/j.tig.2019.11.006 31882191

[B25] Haas B. J. Papanicolaou A. Yassour M. Grabherr M. Blood P. D. Bowden J. (2013). De novo transcript sequence reconstruction from RNA-seq using the Trinity platform for reference generation and analysis. *Nat. Protocols* 8:1494. 10.1038/nprot.2013.084 23845962 PMC3875132

[B26] Haas B. J. Salzberg S. L. Zhu W. Pertea M. Allen J. E. Orvis J. (2008). Automated eukaryotic gene structure annotation using EVidenceModeler and the Program to Assemble Spliced Alignments. *Genome Biol.* 9:R7. 10.1186/gb-2008-9-1-r7 PMC2395244 18190707

[B27] Hoff K. J. Lange S. Lomsadze A. Borodovsky M. Stanke M. (2016). BRAKER1: unsupervised RNA-Seq-Based genome annotation with genemark-ET and AUGUSTUS. *Bioinformatics* 32 767–769. 10.1093/bioinformatics/btv661 26559507 PMC6078167

[B28] Huerta-Espino J. Singh R. P. Germán S. Mccallum B. D. Park R. F. Chen W. Q. (2011). Global status of wheat leaf rust caused by *Puccinia triticina*. *Euphytica* 179 143–160.

[B29] Hurgobin B. Edwards D. (2017). SNP Discovery using a pangenome: has the single reference approach become obsolete? *Biology* 6:21. 10.3390/biology6010021 28287462 PMC5372014

[B30] Jones J. D. G. Dangl J. L. (2006). The plant immune system. *Nature* 444 323–329. 17108957 10.1038/nature05286

[B31] Kämper J. Reichmann M. Romeis T. Bölker M. Kahmann R. (1995). Multiallelic recognition: Nonself-dependent dimerization of the bE and bW homeodomain proteins in ustilago maydis. *Cell* 81 73–83. 10.1016/0092-8674(95)90372-0 7720075

[B32] Kolmer J. A. Hughes M. E. (2016). Physiologic specialization of *Puccinia triticina* on wheat in the United States in 2014. *Plant Dis.* 100 1768–1773. 10.1094/pdis-12-15-1461-sr 30686220

[B33] Krogh A. Larsson B. Von Heijne G. Sonnhammer E. L. L. (2001). Predicting transmembrane protein topology with a hidden markov model: application to complete genomes11Edited by F. Cohen. *J. Mol. Biol.* 305 567–580. 10.1006/jmbi.2000.4315 11152613

[B34] Kronenberg Z. N. Rhie A. Koren S. Concepcion G. T. Peluso P. Munson K. M. (2019). Extended haplotype phasing of &lt;em&gt;de novo&lt;/em&gt; genome assemblies with FALCON-Phase. *bioRxiv [Preprint]* 10.1101/327064 PMC8081726 33911078

[B35] Krzywinski M. Schein J. Birol I. Connors J. Gascoyne R. Horsman D. (2009). Circos: an information aesthetic for comparative genomics. *Genome Res.* 19 1639–1645. 10.1101/gr.092759.109 19541911 PMC2752132

[B36] Kües U. James T. Heitman J. (2011). “6 Mating type in basidiomycetes: unipolar, bipolar, and tetrapolar patterns of sexuality,” in *Evolution of Fungi and Fungal-Like Organisms*, eds Wöstemeyer J. Pöggeler S. (Berlin: Springer), 97–160. 10.1007/978-3-642-19974-5_6

[B37] Kurtz S. Phillippy A. Delcher A. L. Smoot M. Shumway M. Antonescu C. (2004). Versatile and open software for comparing large genomes. *Genome Biol.* 5 R12–R12. 14759262 10.1186/gb-2004-5-2-r12 PMC395750

[B38] Lawrence M. Huber W. Pagès H. Aboyoun P. Carlson M. Gentleman R. (2013). Software for computing and annotating genomic ranges. *PLoS Comput. Biol.* 9:e1003118. 10.1371/journal.pcbi.1003118 23950696 PMC3738458

[B39] Lechner M. Findeiß S. Steiner L. Marz M. Stadler P. F. Prohaska S. J. (2011). Proteinortho: Detection of (Co-)orthologs in large-scale analysis. *BMC Bioinformat.* 12:124. 10.1186/1471-2105-12-124 21526987 PMC3114741

[B40] Lee T.-H. Guo H. Wang X. Kim C. Paterson A. H. (2014). SNPhylo: a pipeline to construct a phylogenetic tree from huge SNP data. *BMC Genomics* 15:162. 10.1186/1471-2164-15-162 24571581 PMC3945939

[B41] Li F. Upadhyaya N. M. Sperschneider J. Matny O. Nguyen-Phuc H. Mago R. (2019a). Emergence of the Ug99 lineage of the wheat stem rust pathogen through somatic hybridisation. *Nat. Commun.* 10:5068. 10.1038/s41467-019-12927-7 PMC6838127 31699975

[B42] Li H. (2018). Minimap2: pairwise alignment for nucleotide sequences. *Bioinformatics* 34 3094–3100. 10.1093/bioinformatics/bty191 29750242 PMC6137996

[B43] Li H. Durbin R. (2009). Fast and accurate short read alignment with Burrows-Wheeler transform. *Bioinformatics* 25 1754–1760. 10.1093/bioinformatics/btp324 19451168 PMC2705234

[B44] Li H. Feng X. Chu C. (2020a). The design and construction of reference pangenome graphs. *arXiv.* doi: 2003.06079v1 10.1186/s13059-020-02168-z PMC7568353 33066802

[B45] Li Y. Xia C. Wang M. Yin C. Chen X. (2019b). Genome sequence resource of a *Puccinia striiformis* isolate infecting wheatgrass. *Phytopathology* 109 1509–1512. 10.1094/phyto-02-19-0054-a 31044663

[B46] Li Y. Xia C. Wang M. Yin C. Chen X. (2020b). Whole-genome sequencing of Puccinia striiformis f. sp. tritici mutant isolates identifies *avirulence* gene candidates. *BMC Genomics* 21:247. 10.1186/s12864-020-6677-y 32197579 PMC7085141

[B47] Liu Y. Du H. Li P. Shen Y. Peng H. Liu S. (2020). Pan-genome of wild and cultivated soybeans. *Cell* 182 162–176.e13. 32553274 10.1016/j.cell.2020.05.023

[B48] Love M. I. Huber W. Anders S. (2014). Moderated estimation of fold change and dispersion for RNA-seq data with DESeq2. *Genome Biol.* 15:550. 10.1186/s13059-014-0550-8 PMC4302049 25516281

[B49] Lowe T. M. Chan P. P. (2016). tRNAscan-SE On-line: integrating search and context for analysis of transfer RNA genes. *Nucleic Acids Res.* 44 W54–W57. 27174935 10.1093/nar/gkw413 PMC4987944

[B50] Maia T. M. Lopes S. T. Almeida J. M. G. C. F. Rosa L. H. Sampaio J. P. Gonçalves P. (2015). Evolution of mating systems in basidiomycetes and the genetic architecture underlying mating-type determination in the yeast *Leucosporidium scottii*. *Genetics* 201 75–89. 10.1534/genetics.115.177717 26178967 PMC4566278

[B51] McKenna A. Hanna M. Banks E. Sivachenko A. Cibulskis K. Kernytsky A. (2010). The genome analysis toolkit: a MapReduce framework for analyzing next-generation DNA sequencing data. *Genome Res.* 20 1297–1303. 10.1101/gr.107524.110 20644199 PMC2928508

[B52] Miller M. E. Zhang Y. Omidvar V. Sperschneider J. Schwessinger B. Raley C. (2018). *De Novo* assembly and phasing of Dikaryotic Genomes from Two Isolates of *Puccinia coronata* f. sp. avenae, the causal agent of oat crown rust. *mBio* 9:e1650–17. 10.1128/mBio.01650-17 PMC5821079 29463655

[B53] Nattestad M. Schatz M. C. (2016). Assemblytics: a web analytics tool for the detection of variants from an assembly. *Bioinformatics (Oxford, England)* 32 3021–3023. 10.1093/bioinformatics/btw369 27318204 PMC6191160

[B54] Nieuwenhuis B. P. S. Billiard S. Vuilleumier S. Petit E. Hood M. E. Giraud T. (2013). Evolution of uni- and bifactorial sexual compatibility systems in fungi. *Heredity* 111 445–455. 10.1038/hdy.2013.67 23838688 PMC3833681

[B55] Park R. F. Burdon J. J. Jahoor A. (1999). Evidence for somatic hybridization in nature in *Puccinia recondita* f. sp. tritici, the leaf rust pathogen of wheat. *Mycol. Res.* 103 715–723. 10.1017/s0953756298007631

[B56] Park R. F. Oates J. D. Meldrum S. (2000). Recent pathogenic changes in the leaf (brown) rust pathogen of wheat and the crown rust pathogen of Oats in Australia in relation to host resistance. *Acta Phytopathol. Entomol. Hungarica* 35 387–394.

[B57] Park R. F. Wellings C. R. (2012). Somatic hybridization in the uredinales. *Ann. Rev. Phytopathol.* 50 219–239. 10.1146/annurev-phyto-072910-095405 22920559

[B58] Pertea M. Kim D. Pertea G. M. Leek J. T. Salzberg S. L. (2016). Transcript-level expression analysis of RNA-seq experiments with HISAT, StringTie and Ballgown. *Nat. Protocols* 11 1650–1667. 10.1038/nprot.2016.095 27560171 PMC5032908

[B59] Prasad P. Savadi S. Bhardwaj S. C. Gangwar O. P. Kumar S. (2019). Rust pathogen effectors: perspectives in resistance breeding. *Planta* 250 1–22. 10.1007/s00425-019-03167-6 30980247

[B60] Rawlings N. D. Barrett A. J. Finn R. (2016). Twenty years of the MEROPS database of proteolytic enzymes, their substrates and inhibitors. *Nucleic Acids Res.* 44 D343–D350. 26527717 10.1093/nar/gkv1118 PMC4702814

[B61] Savary S. Willocquet L. Pethybridge S. J. Esker P. Mcroberts N. Nelson A. (2019). The global burden of pathogens and pests on major food crops. *Nat. Ecol. Evol.* 3 430–439. 10.1038/s41559-018-0793-y 30718852

[B62] Schwessinger B. Chen Y.-J. Tien R. Vogt J. K. Sperschneider J. Nagar R. (2020). Distinct life histories impact Dikaryotic genome evolution in the rust fungus *Puccinia striiformis* causing stripe rust in wheat. *Genome Biol. Evol.* 12 597–617. 10.1093/gbe/evaa071 32271913 PMC7250506

[B63] Schwessinger B. Rathjen J. P. (2017). “Extraction of high molecular weight DNA from fungal rust spores for long read sequencing,” in *Wheat Rust Diseases: Methods and Protocols*, ed. Periyannan S. (New York, NY: Springer New York), 49–57. 10.1007/978-1-4939-7249-4_5 28856640

[B64] Schwessinger B. Sperschneider J. Cuddy W. S. Garnica D. P. Miller M. E. Taylor J. M. (2018). A near-complete haplotype-phased genome of the Dikaryotic Wheat Stripe Rust Fungus *Puccinia striiformis* f. sp. *tritici* Reveals High Interhaplotype Diversity. *mBio* 9:e2275–17. 10.1128/mBio.02275-17 PMC5821087 29463659

[B65] Simao F. A. Waterhouse R. M. Ioannidis P. Kriventseva E. V. Zdobnov E. M. (2015). BUSCO: assessing genome assembly and annotation completeness with single-copy orthologs. *Bioinformatics* 31 3210–3212. 10.1093/bioinformatics/btv351 26059717

[B66] Slater G. S. C. Birney E. (2005). Automated generation of heuristics for biological sequence comparison. *BMC Bioinformatics* 6:31. 10.1186/1471-2105-6-31 15713233 PMC553969

[B67] Song L. Wu J. Q. Dong C. M. Park R. F. (2020). Integrated analysis of gene expression, SNP, InDel, and CNV identifies candidate avirulence genes in Australian isolates of the wheat leaf rust pathogen *Puccinia triticina*. *Genes (Basel)* 11 1107. 10.3390/genes11091107 32967372 PMC7564353

[B68] Tettelin H. Masignani V. Cieslewicz M. J. Donati C. Medini D. Ward N. L. (2005). Genome analysis of multiple pathogenic isolates of Streptococcus agalactiae: implications for the microbial “pan-genome”. *Proc. Natl. Acad. Sci. U.S.A.* 102 13950–13955. 16172379 10.1073/pnas.0506758102 PMC1216834

[B69] Wallen R. M. Perlin M. H. (2018). An overview of the function and maintenance of sexual reproduction in dikaryotic fungi. *Front. Microbiol.* 9:503. 10.3389/fmicb.2018.00503 29619017 PMC5871698

[B70] Wu J. Q. Dong C. Song L. Park R. F. (2020). Long-Read–Based de novo genome assembly and comparative genomics of the wheat leaf rust pathogen *Puccinia triticina* Identifies candidates for three avirulence genes. *Front. Genet.* 11:521. 10.3389/fgene.2020.00521 32582280 PMC7287177

[B71] Wu J. Q. Sakthikumar S. Dong C. Zhang P. Cuomo C. A. Park R. F. (2017). Comparative genomics integrated with association analysis identifies candidate effector genes corresponding to *Lr20* in Phenotype-Paired *Puccinia triticina* Isolates from Australia. *Front. Plant Sci.* 8:148. 10.3389/fpls.2017.00148 28232843 PMC5298990

[B72] Wu T. D. Watanabe C. K. (2005). GMAP: a genomic mapping and alignment program for mRNA and EST sequences. *Bioinformatics* 21 1859–1875. 10.1093/bioinformatics/bti310 15728110

[B73] Xu J. Linning R. Fellers J. Dickinson M. Zhu W. Antonov I. (2011). Gene discovery in EST sequences from the wheat leaf rust fungus *Puccinia triticina* sexual spores, asexual spores and haustoria, compared to other rust and corn smut fungi. *BMC Genomics* 12:161. 10.1186/1471-2164-12-161 21435244 PMC3074555

[B74] Yin Y. Mao X. Yang J. Chen X. Mao F. Xu Y. (2012). dbCAN: a web resource for automated carbohydrate-active enzyme annotation. *Nucleic Acids Res.* 40 W445–W451. 22645317 10.1093/nar/gks479 PMC3394287

[B75] Yu G. Lam T. T.-Y. Zhu H. Guan Y. (2018). Two methods for mapping and visualizing associated data on phylogeny using Ggtree. *Mol. Biol. Evol.* 35 3041–3043. 10.1093/molbev/msy194 30351396 PMC6278858

